# Dermalix enhances angiogenesis and collagen remodeling in an ischemic colon anastomosis rat model

**DOI:** 10.1590/acb413526

**Published:** 2026-06-29

**Authors:** Yasemin Keskin, Alperen Irşat Keskin, Siyar Ersoz, Cevriye Cansız Ersoz, Ilknur Kepenekci Bayram, Ayhan Bülent Erkek

**Affiliations:** 1 Ankara Dr. Abdurrahman Yurtaslan Oncology Training and Research Hospital – Department of General Surgery – Ankara – Turkey.; 2 Ünye State Hospital – Department of General Surgery – Ordu – Turkey.; 3 Ankara University – School of Medicine – Department of General Surgery – Ankara – Turkey.; 4 Ankara University – School of Medicine – Department of Pathology – Ankara – Turkey.

**Keywords:** Colon, Ischemia, Anastomotic Leak, Wound Healing, Angiogenesis

## Abstract

**Purpose:**

To evaluate the effect of Dermalix on anastomotic healing in a rat model of ischemic colon anastomosis.

**Methods:**

Forty rats were divided into three groups: negative control (n = 10), control (n = 15), and experimental (n = 15). Only laparotomy was performed in the negative control group. In the control group, ischemia and end-to-end colon anastomosis were performed, while in the experimental group, Dermalix was applied circumferentially around the anastomosis. On postoperative day 7, anastomotic segments were evaluated for burst pressure, adhesion, and histopathological parameters.

**Results:**

Abscess formation related to anastomotic leakage occurred in two rats in the control group and in one rat in the experimental group. Mean burst pressure was 134.7 mmHg in the control group and 194.7 mmHg in the experimental group (*p* = 0.005). No significant differences were observed in inflammation, epithelial regeneration, or granulation tissue formation. Neovascularization (*p* < 0.001) and collagenization (*p* = 0.032) were significantly higher in the Dermalix group. There was no significant difference in adhesion or foreign body reaction.

**Conclusion:**

Dermalix improved key histological and mechanical indicators of anastomotic healing under ischemic conditions, providing preliminary evidence of its potential as a bioactive adjunct. Further studies are warranted to confirm these findings and assess clinical relevance.

## Introduction

Anastomotic leakage is a common complication in small intestine and colon surgeries, often associated with serious outcomes. Although there is a substantial body of literature examining leakage rates and the associated risk factors for anastomotic leakage, high-quality evidence to guide management approaches is limited^1^. Ischemia, a condition that impairs wound healing, can occur at any stage of intestinal surgery. Given the complications arising from intestinal ischemia, its management is of critical importance.

Considering that anastomotic healing is a form of wound healing, a preliminary study was conducted with the hypothesis that Dermalix (Dx) could accelerate anastomotic healing and prevent leakages. In this preliminary study, the potential effect of Dx on colon anastomoses was investigated^2^. However, its impact under ischemic conditions has not been explored yet.

Based on these findings, this study aimed to evaluate the wound-healing effect of Dx, which had previously been assessed and proven effective on colon anastomoses, specifically on ischemic colon anastomoses. We anticipated that Dx would enhance neovascularization, accelerate wound healing, and improve the mechanical and histological integrity of the anastomotic site under ischemic conditions.

Although the ultimate indicator of clinical success is the presence or absence of anastomotic leakage, the present experimental design primarily focused on surrogate biological parameters—such as bursting pressure and histological remodeling—to elucidate the mechanistic aspects of healing. This preclinical approach aimed to provide a biological explanation for how Dx may contribute to anastomotic reinforcement in ischemic conditions.

## Methods

### Selection of experimental animals

This preclinical study aimed to investigate the effects of Dx on anastomotic healing and the prevention of leakage in a rat model of ischemic colon. The *Rattus norvegicus* species was selected due to its anatomical suitability, manageable size, and physiological similarity to human tissue healing processes. Ethical approval for the study was obtained from the Ankara University Local Animal Ethics Committee (Approval No.: 2023-6-39). The study was conducted in March 2023 at the Animal Laboratory of Ankara University’s Faculty of Medicine.

In total, 40 rats were used in this study (10 negative controls, 15 controls, and 15 in the experimental group), with no perioperative or postoperative animal losses. Group allocation was performed at the cage level: animals that had been reared together were assigned to the same experimental group to minimize postoperative incompatibility or injury. However, the surgical team remained blinded to cage assignment.

Although a formal power analysis was not performed, the sample size was based on our previous pilot study, which yielded statistically significant results using the same experimental design^2^. A post-hoc power analysis was performed for the primary endpoint (bursting pressure). Based on the observed effect size (Cohen’s d ≈ 1.18), the achieved power was ≈ 79% with the current sample size (n = 15 per group). An *a-priori* analysis suggested that 16 animals per group would be required for 80% power. These results indicated that our sample size was borderline but acceptable.

### Dermalix

Dx is a bioactive wound dressing composed of collagen as its fibrous component, the cell-binding protein laminin, and hyaluronic acid (HA), a glycosaminoglycan derivative known to promote angiogenesis and reduce inflammation^3-6^. Dipalmitoylphosphatidylcholine (DPPC), a phospholipid resembling the cell membrane structure, was incorporated into the microparticle matrix to enhance the encapsulation of resveratrol (RSV), a potent antioxidant that stimulates fibroblast activity and collagen synthesis^4,7^. The product was specifically engineered to contain microparticles larger than 20 µm, ensuring sustained RSV release during wound healing and minimizing rapid phagocytosis-associated scar formation^7,8^. This synergistic RSV–HA system was developed to strengthen antioxidant effects and maintain the delicate wound microenvironment and has previously shown favorable outcomes in diabetic models of acute and chronic wound healing^3^.

### Surgical procedure

**Figure 1:**
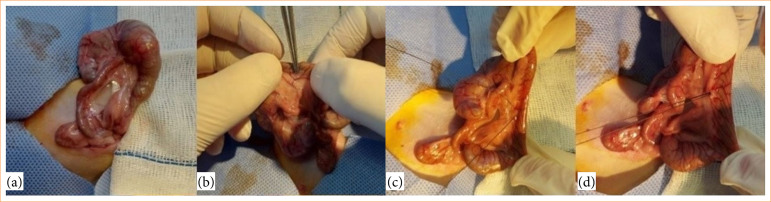
Experimental model of ischemic colon anastomosis. (a) Normal appearance of the rat colon. (b) Vascular arcade supplying the segment planned for anastomosis, indicated with forceps. (c) Elevation of the vascular arcade prior to ligation. (d) Ligation of the ileocolic artery.

Source: Elaborated by the authors.

All procedures were performed under sterile conditions and general anesthesia (ketamine 90 mg/kg, intramuscularly). The experiments were performed in a fixed sequence (negative control, experimental, and control groups). A midline laparotomy was performed in all animals. In the control and experimental groups, the ileocolic artery was identified and ligated to create segmental ischemia of the right colon, adapted from the ischemia model of Rodriguez-Leon et al.^9^ (Fig. 1).

Ligation of the ileocolic artery resulted in a segmental ischemia involving approximately 2–3 cm of the right colon, confirmed visually by color change and reduced peristalsis. The end-to-end anastomosis was performed using 6/0 Prolene sutures, placing 8–10 interrupted stitches within the ischemic segment, approximately 1–2 cm distal to the ligated ileocolic artery, to simulate compromised perfusion conditions.

In the experimental group, after completing the anastomosis, a 1×1-cm piece of Dx was wrapped circumferentially around the anastomotic site with the suture line centered. The abdominal wall was then closed in anatomical layers in all groups. In the negative control group, only laparotomy was performed without any ischemia or anastomosis.

The primary endpoint was the effect of Dx on mechanical anastomotic strength, evaluated by bursting pressure measurements. The secondary endpoint was its effect on histopathological wound healing, including angiogenesis, fibroblast proliferation, and collagen deposition.

Although the experimental setup was not designed to assess clinical leakage as a powered outcome, any presence of intra-abdominal abscess, peritonitis, or overt leakage observed during relaparotomy was recorded as an exploratory finding related to anastomotic integrity.

No inert biomaterial comparator was used, because Dx was the only available bioactive dressing in our laboratory at the time of the study. Therefore, the control and experimental groups were designed to evaluate the biological contribution of Dx to ischemic anastomotic healing, rather than to assess biomaterial specificity.

### Postoperative monitoring and tissue collection

All animals received standard laboratory care in accordance with institutional ethical guidelines. They were housed under controlled environmental conditions (12 h light/dark cycle, temperature 22 ± 2°C, humidity 50–60%) with free access to water and standard chow, except during the immediate postoperative phase. Postoperatively, animals were fasted for the first 24 hours, provided with water only between 24–48 hours, and subsequently allowed free access to both food and water. Animals were monitored daily throughout the study, and no signs of pain, distress, or abnormal behavior were observed. No differences in clinical behavior were noted among the experimental groups.

On postoperative day 7, a second laparotomy was performed under anesthesia. In the control and experimental groups, a 3-cm colonic segment including the anastomosis was resected. Euthanasia was performed by thoracic aorta incision under deep anesthesia.

### Adhesion scoring

Adhesions were assessed macroscopically using the scoring system developed by Van der Ham et al.^10^, which evaluates the extent and severity of adhesions between the anastomosis site, omentum, and adjacent intestinal structures.

### Burst pressure measurement

After resection, a 1-cm colonic segment containing the anastomosis in the center was isolated for burst pressure testing, as described in our previous protocol^2^. Bursting pressure measurements were performed by a different surgeon, also blinded to the experimental groups.

### Histopathological evaluation

Outcome assessment was conducted under blinded conditions. Histopathological evaluation was conducted by an independent pathologist who was not present during the procedures; tissue samples were sequentially numbered (1–30) and submitted without information on group allocation.

Following pressure testing, tissue segments were opened along the mesenteric border, rinsed with 0.9% saline, and fixed in 10% buffered formalin. After routine histological processing, 3-mm sections including the anastomotic line were embedded in paraffin. Four-micron sections were stained with hematoxylin-eosin (H&E) and evaluated under light microscopy.

Histological parameters assessed included neutrophilic infiltration, epithelial regeneration, foreign body reaction, granulation tissue formation, local inflammation, and neovascularization. Each parameter was graded semi-quantitatively as mild (1), moderate (2), or severe (3). To evaluate collagenization, Masson’s trichrome staining was used and similarly scored.

### Statistical analysis

Categorical variables were presented as numbers and percentages. Continuous variables were expressed as mean ± standard deviation or median with range, depending on data distribution. The Pearson’s χ^2^ test was used for categorical comparisons, while the Mann–Whitney’s U test was used for continuous variables. Statistical analyses were performed using Statistical Package for the Social Sciences 23.0 and GraphPad Prism 8.0 software.

To account for multiple comparisons among histological parameters, p-values were adjusted using the Benjamini–Hochberg false discovery rate (FDR) correction. Effect sizes (Cohen’s d for continuous variables) were also calculated to evaluate the magnitude of differences between groups. A q-value (FDR-adjusted *p*) < 0.05 was considered statistically significant.

## Results

On the seventh postoperative day, limited abscess formation associated with anastomotic leakage was observed in two rats in the control group and one rat in the experimental group. In rats with perforation, the abdominal cavity showed signs of peritonitis, such as a congested appearance and increased temperature (Table 1).

**Table 1:** Comparison of mechanical and clinical outcomes between the control and Dermalix groups.

Parameter	Control group	Dermalix group	*p* -/q-value
Total animals, n	15	15	-
Anastomotic leakage, n (%)	2 (13.3)	1 (6.7)	-
Animals analyzed for burst pressure, n	13	14	-
Burst pressure (mmHg), mean ± standard deviation	134.7 ± 67.7	194.7 ± 66.6	q = 0.034
Adhesion score	Similar between groups	Similar between groups	*p* = 0.70

Source: Elaborated by the authors.

Adhesion development was observed in both groups, but there was no statistically significant difference between the control and experimental groups in terms of presence or severity of adhesions (*p* = 0.70, Pearson’s χ^2^ test) (Table 1).

**Figure 2:**
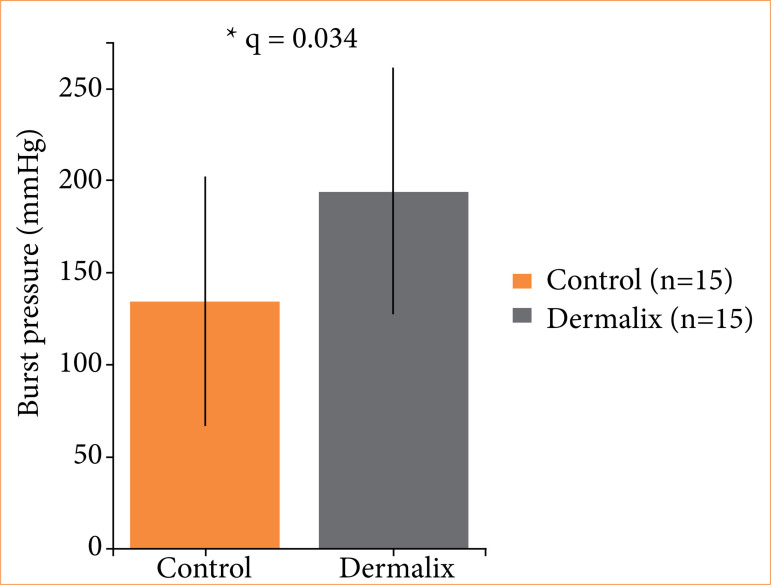
Comparison of anastomotic bursting pressure between control and Dermalix groups. Bars represent mean ± standard deviation. q = 0.034 (FDR-corrected Mann–Whitney’s U test).

Source: Elaborated by the authors.

The first parameter used to evaluate anastomotic healing was burst pressure, an indicator of mechanical strength. Due to anastomotic leakage, burst pressure measurements could not be obtained from two rats in the control group and one rat in the experimental group. In the remaining rats, burst pressure ranged between 78 and 220 mmHg in the control group (mean ± standard deviation = 134.7 ± 67.7 mmHg) and between 100 and 260 mmHg in the experimental group (mean ± standard deviation = 194.7 ± 66.6 mmHg). The difference between the two groups was statistically significant in favor of the experimental group (q = 0.034, FDR-corrected Mann–Whitney’s U test) (Table 1, Fig. 2).

The second parameter was histopathological evaluation. In both groups, neutrophilic infiltration, local inflammation, epithelial regeneration, and granulation tissue formation were assessed. No statistically significant differences were observed between the control and experimental groups in these parameters (q > 0.05 for all, FDR-corrected Mann–Whitney’s U test) (Table 2).

**Table 2:** Histopathological findings.

Parameter	Control group	Dermalix group	q-value
Neutrophilic infiltration	No significant difference	No significant difference	> 0.05
Local inflammation	No significant difference	No significant difference	> 0.05
Epithelial regeneration	No significant difference	No significant difference	> 0.05
Granulation tissue formation	No significant difference	No significant difference	> 0.05
Neovascularization	Mild–Moderate predominant	Moderate–Severe predominant	0.001
Collagenization	Mild–Moderate	Severe predominant	0.028

Source: Elaborated by the authors.

**Figure 3:**
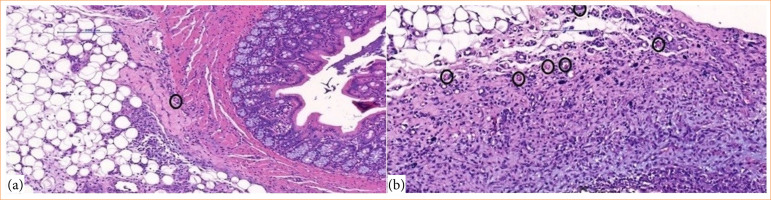
Histopathological evaluation of the anastomotic site. (a) Control group (hematoxylin and eosin stain, ×100 magnification). (b) Experimental group (hematoxylin and eosin stain, ×200 magnification).

Source: Elaborated by the authors.

**Figure 4:**
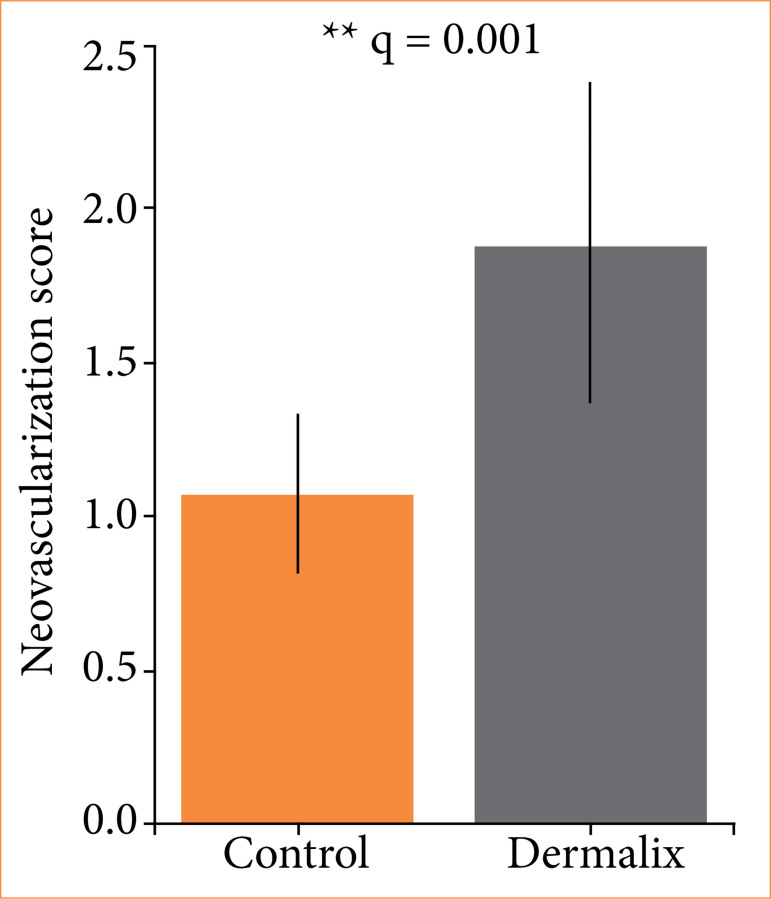
Comparison of neovascularization scores between groups. Bars represent mean ± standard deviation. q = 0.001.

Source: Elaborated by the authors.

Regarding neovascularization, moderate to severe new vessel formation was detected in most rats in the experimental group, while severe neovascularization was absent in the control group (Fig. 3). The difference in neovascularization scores between the groups was statistically significant (q = 0.001, FDR-corrected Mann–Whitney’s U test) (Table 2, Fig. 4).

**Figure 5:**
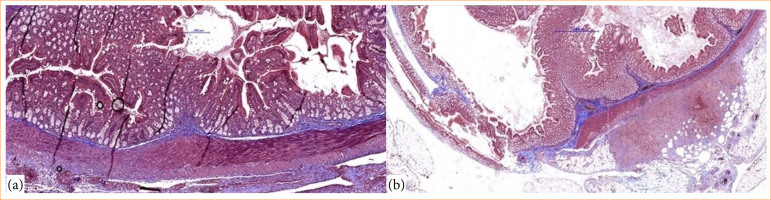
Collagen deposition at the anastomotic site. (a) Control group (Masson’s trichrome stain, ×50 magnification). (b) Experimental group (Masson’s trichrome stain, ×20 magnification).

Source: Elaborated by the authors.

**Figure 6:**
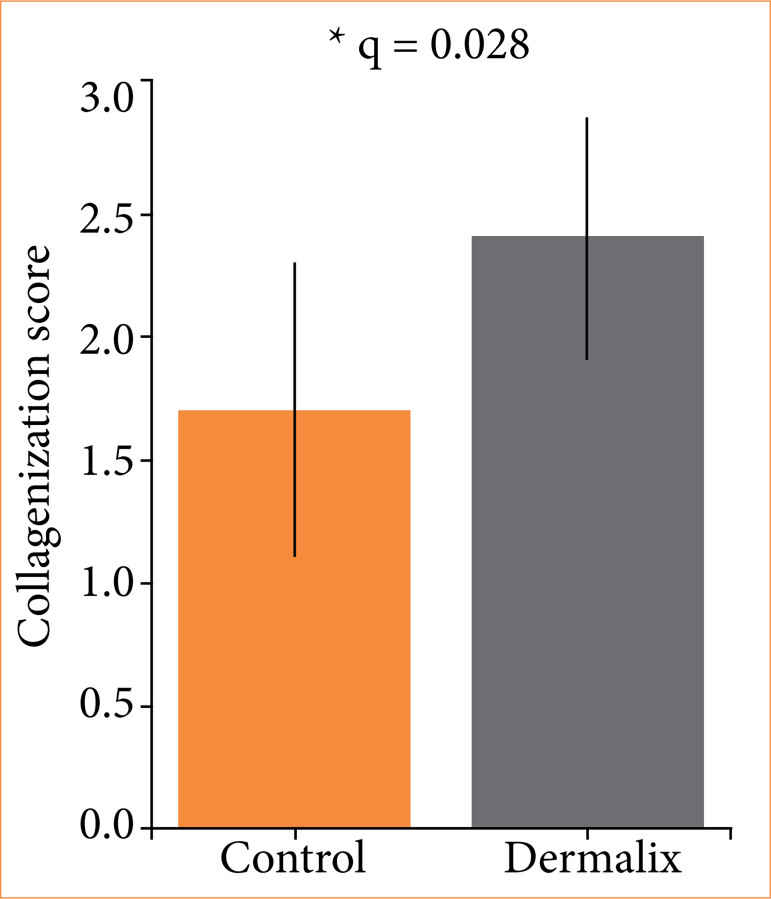
Comparison of collagenization scores between groups. Bars represent mean ± standard deviation. q = 0.028.

Source: Elaborated by the authors.

Collagenization, evaluated using Masson’s trichrome staining, showed a consistent increase in the experimental group. Mild to moderate collagen deposition was observed in the control group, while severe collagenization was detected only in the experimental group (Fig. 5). The difference between groups was statistically significant (q = 0.028, FDR-corrected Mann–Whitney’s U test) (Table 2, Fig. 6).

No significant correlations were found between foreign body reaction scores and adhesion scores (*p* = 0.91, Pearson’s χ^2^ test). These findings indicated that Dx primarily enhances mechanical and histological healing parameters through improved vascularization and collagen remodeling rather than by altering inflammatory or adhesion responses.

## Discussion

In this experimental model of ischemic colon anastomosis, the application of Dx significantly increased anastomotic bursting pressure compared to the control group. Histopathological evaluation demonstrated significantly enhanced neovascularization and collagen deposition in the experimental group, while no significant differences were observed in inflammatory parameters, epithelial regeneration, or adhesion formation. Anastomotic leakage occurred in 13.3% of the control group and 6.7% of the experimental group.

The healing of intestinal anastomoses remains a major concern in colorectal surgery, particularly in the presence of ischemia. Anastomotic leakage is one of the most feared complications and is associated with significant morbidity and mortality, with reported incidence rates ranging from 1 to 26%^11^. Although the present study was not powered for leak rate comparison, the observed leakage rates provide contextual information regarding anastomotic integrity within this experimental model.

Anastomotic healing is generally divided into two phases. The early phase (days 1–3) is characterized by low mechanical strength, while the proliferative phase (days 4–15) is marked by collagen synthesis and an increase in tissue strength^12,13^. Based on previous data, we chose postoperative day 7 as the endpoint of our study to evaluate anastomotic integrity. At this time point, we observed a significant increase in burst pressure (q = 0.034, FDR-corrected) in the experimental group compared to the control group. This suggests that the Dx product positively contributed to mechanical healing. The pressure values in this study were also higher than those observed in our previous pilot study^2^, further supporting the findings. The higher mean bursting pressures in this study compared to our pilot study can be explained by improved experience in animal handling and surgical procedures, highlighting the value of a pilot study.

Although wound healing is often studied in the context of skin, the gastrointestinal tract exhibits its own unique phases of inflammation, proliferation, and maturation^14^. In the inflammatory phase, neutrophils migrate to the wound site and reach peak levels within 12 to 24 hours14. The lack of significant differences in neutrophil infiltration between the groups may be attributed to the timing of evaluation on postoperative day 7, by which neutrophilic activity typically subsides. Consistent with the findings of the present study, Kayapınar et al.^15^ reported no significant difference in neutrophil infiltration on postoperative day 7 in an experimental model of ischemic intestinal anastomosis treated with intraperitoneal carbon monoxide–releasing molecule after the induction of ischemia.

Following the initial inflammatory response, macrophages and fibroblasts become predominant at the wound site, initiating collagen synthesis and neovascularization. Early fibroblast proliferation facilitates new collagen deposition, which is crucial for restoring anastomotic strength^16,17^. By day 3 after anastomosis, collagenase activity increases substantially, leading to a reduction in suture retention by up to 80%—a phenomenon known as the delay phase^18^. Persistent inflammation during this period may impair healing and increase the risk of leakage^19^.

Dx, which contains RSV, has been shown to protect tissues from proteolytic enzymes and stimulate fibroblast proliferation and collagen synthesis^7^. This could explain the enhanced collagenization (q = 0.028, FDR-corrected) and higher burst pressures observed in the experimental group. Additionally, HA, another component of Dx, has been reported to enhance fibroblast function while reducing neutrophilic infiltration and tissue necrosis^20,21^.

Collagen-based materials are widely utilized in surgical practice due to their biocompatibility and ability to integrate with host tissues^22,23^. The combination of HA, RSV, and collagen in Dx appears to provide synergistic support for wound healing. In this study, collagenization was significantly more pronounced in the experimental group, which may have contributed to better anastomotic integrity. This supports the mechanistic effect of Dx on matrix remodeling rather than a direct clinical endpoint such as leak prevention. Histologically, collagen replaces the initial fibrin matrix, strengthening the anastomotic site and potentially reducing the risk of dehiscence. These findings highlight the biological reinforcement role of Dx, which may serve as a foundation for future translational studies focusing on leak outcomes.

Similar findings have been reported in other experimental studies evaluating biological agents that enhance anastomotic healing. In an experimental peritonitis model, the application of fibroblast growth factor–containing collagen and antibiotic collagen was shown to increase fibroblastic activity, neovascularization, and tissue hydroxyproline levels compared with control groups^24^. Although the increase in bursting pressure did not reach statistical significance in that study, the enhanced collagen synthesis and vascular proliferation suggested improved wound healing capacity. Consistent with these observations, our study demonstrated significantly increased collagen deposition and neovascularization in the Dx group, supporting the role of biologically active matrices in promoting tissue remodeling and anastomotic strength.

Beyond mechanical reinforcement, epithelial regeneration is also critical for sealing the anastomosis. In both groups, epithelial proliferation and granulation tissue formation were observed without significant differences, indicating that mucosal repair had progressed sufficiently by day 7 to close the luminal defect.

Ischemia is a key contributor to anastomotic failure, and neovascularization plays a fundamental role in overcoming hypoxic injury. Our ischemia model, created by ligation of the ileocolic artery, mimics clinical scenarios involving compromised perfusion. Although mild neovascularization was noted in the control group, the experimental group demonstrated significantly enhanced neovascular response (q = 0.001, FDR-corrected), likely due to the regenerative effects of Dx and its components^25,26^.

RSV is known to promote vascular health by reducing oxidative stress, inhibiting inflammation, and enhancing nitric oxide synthesis in endothelial cells^27^. These effects may have contributed to the pronounced neovascularization observed in the experimental group, supporting oxygenation, collagen production, and epithelial repair. Improved vascularization may thus lower leakage risk and support overall healing in ischemic anastomoses.

Similarly, previous experimental studies have demonstrated that impaired perfusion negatively affects anastomotic healing by reducing fibroblast activity, neovascularization, and collagen synthesis. In a rat model of obstructive ileus, decreased bursting pressure was associated with reduced fibroblast numbers, lower hydroxyproline concentrations, and impaired neovascularization at the anastomotic site^28^. These findings emphasize the critical role of vascularization and extracellular matrix remodeling in maintaining anastomotic integrity. In this context, the increased neovascularization and collagen deposition observed in the present study may represent key mechanisms through which Dx enhances anastomotic healing under ischemic conditions.

In clinical practice, surgeons generally avoid constructing anastomoses in overtly necrotic bowel segments. However, complete ischemia is not the only clinically relevant scenario. Anastomoses are frequently performed under suboptimal perfusion conditions, such as in emergency surgery, oncologic resections after vascular compromise, or in patients with systemic hypoperfusion. The present model was designed to reproduce a controlled ischemic challenge to the anastomotic site, thereby mimicking high-risk physiological conditions rather than absolute tissue necrosis. From this perspective, the experimental setting provides a translational framework to evaluate biological reinforcement strategies in compromised but potentially viable intestinal tissue.

Although the study lacked an inert biomaterial control group, the design aimed to explore the intrinsic biological effects of Dx rather than to distinguish material-specific reactions. Therefore, the results should be interpreted in the context of a mechanistic proof-of-concept study rather than as definitive clinical evidence.

Although foreign body reactions are often linked to adhesion formation, our study found no significant difference in adhesion scores between the groups. Mild foreign body reactions were more frequent in the experimental group, suggesting that Dx is generally well-tolerated. The absence of severe inflammatory or fibrotic reactions supports its potential safety in intestinal surgical applications.

This study has several limitations. Although the rat model provides useful experimental insights, it does not fully replicate the complexity of human physiology. Quantitative biochemical assessment of collagen content, such as hydroxyproline measurement, was not performed, but semi-quantitative histopathological evaluation using Masson’s trichrome staining allowed comparative assessment of collagen deposition between groups. In addition, collagen subtype analysis and ultrastructural evaluation of collagen fibers were not conducted. The relatively short follow-up period limited the evaluation of

long-term outcomes such as stricture formation or delayed leakage. Furthermore, the absence of an inert biomaterial control group and the relatively small sample size restrict the interpretation of foreign body reactions and secondary outcomes. Therefore, larger experimental studies incorporating biochemical and ultrastructural analyses are needed to further clarify the mechanisms underlying anastomotic healing.

## Conclusion

Anastomotic healing is influenced by several factors, including tissue perfusion, surgical technique, and local biological conditions. In this experimental model of ischemic colon anastomosis, the application of Dx significantly improved healing outcomes. The experimental group demonstrated higher burst pressures and enhanced histological features such as neovascularization and collagen deposition, without increased adhesion or severe inflammatory reactions.

These results provided mechanistic evidence that Dx enhances tissue repair and remodeling under ischemic conditions rather than directly proving clinical leak reduction. The findings therefore support the potential of Dx as a bioactive adjunct for improving anastomotic healing, especially in compromised or hypoperfused tissues.

However, due to the absence of an inert comparator material and the exploratory design of this study, the conclusions should be interpreted as preliminary and mechanistic rather than clinical. Further studies with larger sample sizes, extended follow-up, and comparison to other biological agents are needed to confirm these results and explore the clinical applicability of Dx in gastrointestinal surgery.

## Data Availability

All data generated or analyzed during this study are included in this article. Further enquiries can be directed to the corresponding author.
